# Extraction of a specimen through an umbilical zigzag incision during laparoscopic surgery for endometrial cancer

**DOI:** 10.1186/s12957-017-1180-x

**Published:** 2017-05-30

**Authors:** Kazuyoshi Kato, Tsuyoshi Hisa, Maki Matoda, Hidetaka Nomura, Hiroyuki Kanao, Kuniko Utsugi, Nobuhiro Takeshima

**Affiliations:** Department of Gynecology, Cancer Institute Hospital, 3-8-31 Ariake, Koutou-ku, Tokyo, 135-8550 Japan

**Keywords:** Endometrial cancer, Laparoscopic surgery, Specimen extraction, Umbilical zigzag incision

## Abstract

**Background:**

Though laparoscopic surgery has recently been applied in the treatment of early-stage endometrial cancer, the presence of a large uterus is a hindrance to specimen extraction from the abdominal cavity. We describe a laparoscopic surgical technique for endometrial cancer involving the extraction of the resected specimen through an umbilical zigzag incision.

**Case presentation:**

A 63-year-old woman with endometrial cancer underwent a total hysterectomy and bilateral salpingo-oophorectomy that was performed laparoscopically. The surgical specimen was extracted through an umbilical zigzag incision. This umbilical zigzag incision created a larger fascial and peritoneal opening, facilitating the removal of the specimen. The final histopathologic results revealed stage 1A G1 endometrioid adenocarcinoma and multiple uterine leiomyomas. Three months after surgery, the wound in the umbilical region was inconspicuous, along with the inward movement of the umbilicus.

**Conclusions:**

A laparoscopic surgical technique for endometrial cancer involving the extraction of the specimen through an umbilical zigzag incision seems to reduce the difficulties associated with laparoscopic surgery and maintains cosmesis. Further analyses involving larger numbers of cases and long-term follow-up periods are warranted to evaluate this surgical method.

## Background

Today, laparoscopic surgery is used in the treatment of early-stage endometrial cancer as an alternative to laparotomic approaches. The laparoscopic approach has multiple advantages, including less blood loss, a reduction in the need for postoperative analgesics, lower morbidity, quicker recovery, and shorter hospital stay [1]. In addition, no significant differences in recurrence and survival have been observed between endometrial cancer patients treated with laparoscopic surgery and those treated with a laparotomy [2]. Despite the advantages of laparoscopy, the presence of a large uterus is a hindrance to laparoscopic surgery because of the limited operative field, restricted range of instrument motion, and difficulty removing the specimen. Though large uterine tumors can be dissected laparoscopically, specimen extraction from the abdominal cavity frequently requires the enlargement of the port-site incision or the addition of a mini-laparotomy, which can compromise the concept of a minimally invasive surgery [3].

Recently, an umbilical zigzag incision method was developed to create larger fascial and peritoneal openings [4]. This incision can be used to enlarge the umbilical opening, facilitating the removal of the specimens. Here, the authors have applied this technique to laparoscopic surgery for endometrial cancer.

In this report, we describe our experiences with laparoscopic surgery for endometrial cancer involving the extraction of the specimen through an umbilical zigzag incision.

## Case presentation

**Fig. 1:**
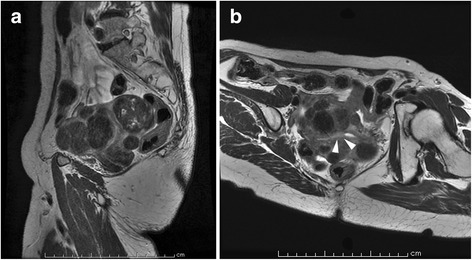
Preoperative magnetic resonance imaging findings of the pelvis show a 2-cm tumor in the uterine cavity (*arrowheads*) and multiple myoma nodules in the uterine corpus

A 63-year-old woman, gravida 3 para 1, presented with complaints of abnormal genital bleeding. A G1 endometrioid adenocarcinoma was identified by an endometrial biopsy. She was then referred to our hospital for the treatment of endometrial cancer. Preoperative magnetic resonance imaging demonstrated a 2-cm tumor in the uterine cavity and multiple myoma nodules in the uterine corpus (Fig. 1). No other lesions, either distant metastasis or nodal metastasis, were observed on an enhanced computed tomography examination.

**Fig. 2:**
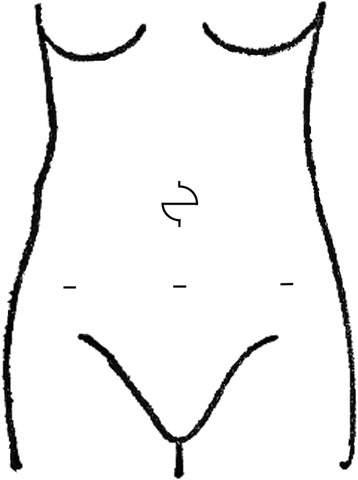
Schema showing the sites of trocar access and the location of the zigzag incision in the umbilical region

**Fig. 3:**
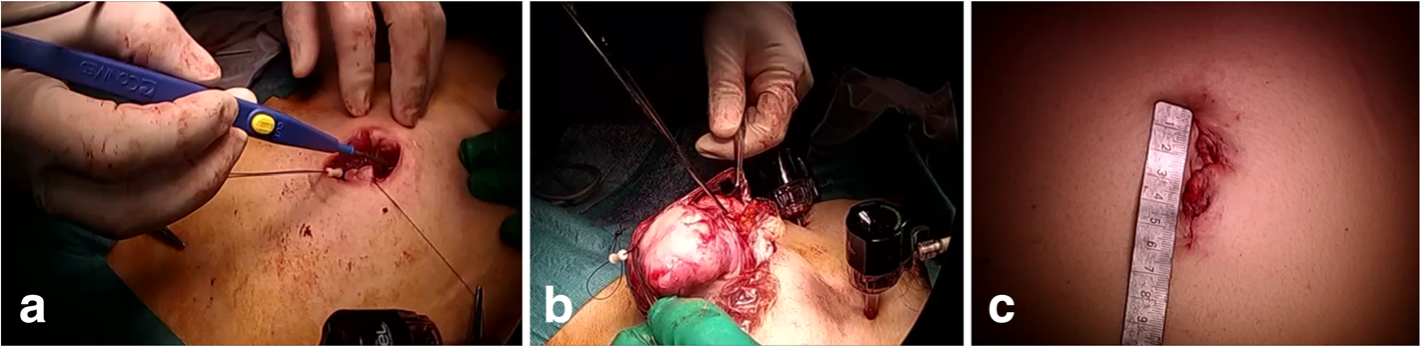
**a** Appearance of making the zigzag incision in the umbilical region. **b** Appearance of the specimen extraction through an umbilical zigzag incision. **c** Appearance of the umbilical region after fascia and skin closure of the incision

**Fig. 4:**
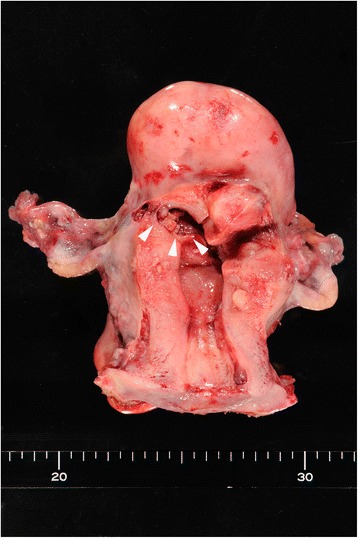
Hysterectomy and bilateral salpingo-oophorectomy specimen. A polypoid mass in the uterine cavity (*arrowheads*) and multiple myoma nodules in the uterine corpus can be seen

During the first step of surgery, a 10-mm trocar was inserted in the umbilicus region and a pneumoperitoneum was established. Two 5-mm trocars were then positioned along bilateral lines between the anterior superior iliac spine and the umbilicus. Another 5-mm trocar was then placed between these two 5-mm trocars (Fig. 2). A total hysterectomy and bilateral salpingo-oophorectomy were laparoscopically performed with these trocars and a vaginal pipe. The surgical specimen was placed into a plastic bag within the abdomen. Because the specimen was too large to be extracted from the abdominal cavity via the vagina, we decided to make a zigzag incision in the umbilicus to remove the entire uterus intact. After making a zigzag skin incision in the umbilicus, the fascial opening was incised along this line, and the diameter of the opening could then be stretched to 6 cm (Fig. 3a, c). Specimen removal was possible through the umbilical zigzag incision without cutting the specimen into smaller pieces (Fig. 3b). The size of the uterus was 8.0 × 7.0 × 12.0 cm in diameter (Fig. 4).

**Fig. 5:**
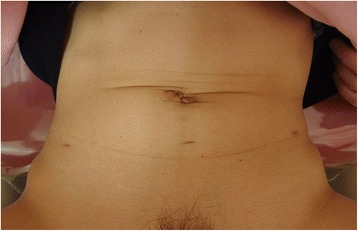
The wound in the umbilical region was inconspicuous at 3 months post-surgery

A histopathological examination revealed an International Federation of Gynecology and Obstetrics (FIGO) stage IA, G1 endometrioid adenocarcinoma located in the uterine fundus measuring 2.5 × 2.0 × 0.5 cm without myometrial invasion. The multiple nodules seen in the uterine corpus were benign leiomyomas. No intra- or early postoperative complications occurred. The postoperative hospital stay was 5 days, which is the conventional stay after a total laparoscopic hysterectomy in our hospital. Three months after surgery, the umbilical incision was inconspicuous, along with the inward movement of the umbilicus (Fig. 5).

## Discussion

We present a surgical technique for the extraction of a specimen through an umbilical zigzag incision during laparoscopic surgery for endometrial cancer.

Severe vaginal atrophy, nulliparity, and enlarged uterus are the most important reasons why the specimen cannot be extracted from the abdominal cavity using the vagina as the optimal route [5]. During surgery for endometrial cancer, these clinical features are often observed. Though the patient in this report was not nulliparous, her uterus was too large to be extracted from the abdominal cavity through the vagina.

Some preliminary reports on the morcellation of enlarged uterine malignant tumors have been made [3, 6]. In these cases, the edge of the bag in which the specimen was to be placed was pulled up to the vaginal introitus, and all the vaginal walls were covered. Uterus morcellation was then performed inside the pouch. The authors concluded that vaginal morcellation is a feasible method of reducing the chance of neoplastic cell spillage. However, the removal of a cancer specimen in pieces is likely to be unacceptable to a notable number of gynecologic oncologists. The Society of Gynecologic Oncology suggests that it is generally contraindicated to morcellate a specimen in the presence of a documented malignancy or in a patient in whom a malignancy is strongly suspected secondary to the potential dissemination into the abdominal cavity [https://www.sgo.org/newsroom/position-statements-2/morcellation/].

Since a preoperative evaluation had suggested a low-risk, early endometrial cancer, we did not perform a systematic lymphadenectomy. Pelvic lymph nodes represent the most common site of extrauterine metastases in patients with clinical early-stage endometrial cancer. Endometrial cancer patients preoperatively diagnosed with a tumor diameter greater than 2.0 cm or with G3 endometrioid or non-endometrioid carcinoma reportedly have a substantial risk (greater than 10%) of lymphatic involvement [7, 8]. We usually recommend that patients with a high risk of lymphatic spread undergo a systematic lymphadenectomy.

Here, we utilized a recently reported surgical technique consisting of an umbilical zigzag incision in laparoscopic surgery for endometrial cancer. This surgical technique was developed to create a larger fascial and peritoneal opening to facilitate the removal of specimens [4]. In the present case with endometrial cancer and multiple leiomyoma nodules, the uterus was successfully extracted in its entirety without hindering the histopathological examination. In addition, the umbilical zigzag incision technique can maintain cosmesis.

## Conclusions

This preliminary report shows that a laparoscopic surgical technique for endometrial cancer involving the extraction of the specimen through an umbilical zigzag incision seems to reduce the difficulties associated with laparoscopic surgery and maintains cosmesis. Further analyses of a larger number of cases and with long-term follow-up are warranted to evaluate this surgical method.

## References

[1] Walker JL, Piedmonte MR, Spirtos NM, Eisenkop SM, Schlaerth JB, Mannel RS (2009). Laparoscopy compared with laparotomy for comprehensive surgical staging of uterine cancer: Gynecologic Oncology Group Study LAP2. J Clin Oncol.

[2] Wright JD, Burke WM, Tergas AI, Hou JY, Huang Y, Hu JC (2016). Comparative effectiveness of minimally invasive hysterectomy for endometrial cancer. J Clin Oncol.

[3] Favero G, Anton C, Silva E, Silva A, Ribeiro A, Araújo MP, Miglino G (2012). Vaginal morcellation: a new strategy for large gynecological malignant tumor extraction: a pilot study. Gynecol Oncol.

[4] Hachisuka T, Kinoshita T, Yamakawa T, Kurata N, Tsutsuyama M, Umeda S (2012). Transumbilical laparoscopic surgery using GelPort through an umbilical zigzag skin incision. Asian J Endosc Surg.

[5] Condous G, Bignardi T, Alhamdan D, Van Calster B, Van Huffel S, Timmerman D (2009). What determines the need to morcellate the uterus during total laparoscopic hysterectomy?. J Minim Invasive Gynecol.

[6] Montella F, Riboni F, Cosma S, Dealberti D, Prigione S, Pisani C (2014). A safe method of vaginal longitudinal morcellation of bulky uterus with endometrial cancer in a bag at laparoscopy. Surg Endosc.

[7] Bogani G, Dowdy SC, Cliby WA, Ghezzi F, Rossetti D, Frigerio L (2016). Management of endometrial cancer: issues and controversies. Eur J Gynaecol Oncol.

[8] Bogani G, Dowdy SC, Cliby WA, Ghezzi F, Rossetti D, Mariani A (2014). Role of pelvic and para-aortic lymphadenectomy in endometrial cancer: current evidence. J Obstet Gynaecol Res.

